# Exploiting holistic approaches to model specificity in protein phosphorylation

**DOI:** 10.3389/fgene.2014.00315

**Published:** 2014-09-30

**Authors:** Antonio Palmeri, Fabrizio Ferrè, Manuela Helmer-Citterich

**Affiliations:** Department of Biology, Centre for Molecular Bioinformatics, University of Rome Tor VergataRome, Italy

**Keywords:** kinase-substrate specificity, phosphorylation context, phosphorylation prediction, cellular signaling, kinase-peptide specificity, substrate recruitment, signaling networks

## Abstract

Phosphate plays a chemically unique role in shaping cellular signaling of all current living systems, especially eukaryotes. Protein phosphorylation has been studied at several levels, from the near-site context, both in sequence and structure, to the crowded cellular environment, and ultimately to the systems-level perspective. Despite the tremendous advances in mass spectrometry and efforts dedicated to the development of *ad hoc* highly sophisticated methods, phosphorylation site inference and associated kinase identification are still unresolved problems in kinome biology. The sequence and structure of the substrate near-site context are not sufficient alone to model the *in vivo* phosphorylation rules, and they should be integrated with orthogonal information in all possible applications. Here we provide an overview of the different contexts that contribute to protein phosphorylation, discussing their potential impact in phosphorylation site annotation and in predicting kinase-substrate specificity.

## Introduction

Phosphorylation, the enzymatic reaction resulting in the addition of a phosphate group to several types of residues, which in eukaryotes are mainly serines, threonines, or tyrosines, generates *de facto* a new side chain whose physico-chemical properties are different from those of the unmodified residues. This mechanism of Post-Translational Modification (PTM) is strikingly common throughout evolution and in particular for eukaryotes where it is involved in a myriad of cellular processes (Manning et al., 2002a,b, 2008, 2011; Caenepeel et al., 2004; Bradham et al., 2006).

The chemical properties of phosphate make this group a perfect candidate for protein modification, and allow its broad use as a molecular switch within the cell (Hunter, 2012). Indeed the hydrolytic stability of phosphate esters (for instance phosphoserine, phosphotyrosine, phosphothreonine, etc.) in aqueous solutions at pH7 allows the cell to minimize the noise in signal transduction due to non-enzymatically catalyzed hydrolysations. In addition, phosphate monoesters act as sensors, as their electric charge can be influenced by the chemical environment. Lastly, phosphate is a largely available molecule, as it is abundant on Earth and particularly within the cell, where it is included in a fundamental energy storage molecule, i.e., ATP. Differently from other types of PTMs, only one group can be enzymatically added to one residue, underlining the peculiar binary nature of this protein modification. The modified residue can undergo inter- or intra-molecular interactions, causing changes to the protein structure or interfering with its function, probably the most famous and complex example being the allosteric regulation of glycogen phosphorylase (Barford et al., 1991). Additional mechanisms for phosphorylation-mediated modulation have also been reported, such as for instance the inhibition of a binding site (Hurley et al., 1990). A beautiful electrostatic-based tuning of protein function mediated by phosphorylation has been described in yeast cell-cycle regulation, where the membrane localization of the MAPKs scaffold protein Ste5 is disrupted by phosphorylation of a cluster of sites flanking a basic membrane binding motif (Strickfaden et al., 2007).

However, the reason for the success of this type of PTM during evolution, at least in eukaryotes, has to be found largely in its ability to be edited and recognized selectively by specific protein domains, thus providing an efficient tool for transient molecular recognition in the context of signal transduction networks (Lim and Pawson, 2010).

With PTM-based proteomics, phosphorylation sites, as well as other PTMs, are identified and stored in large-scale datasets (Olsen and Mann, 2013). As a consequence of this explosion of data, there is great demand for functional annotation studies that largely exceeds what current technology offers. Furthermore, some observations question the functionality of a substantial fraction of these sites (Landry et al., 2009; Moses and Landry, 2010; Levy et al., 2012; Tan and Bader, 2012).

Given the difficulties in the experimental annotation of the kinase responsible for the phosphorylation, many attempts have been made to computationally model cellular signaling events. Some of the published reviews examine the field of kinase specificity from a more biological perspective, discussing the protein kinase specificity rules in sequence and in structure, while some others compare the different tools, and the techniques used to model kinase-substrate interaction and in general those used to build phosphorylation site predictors (Zhu et al., 2005; Ubersax and Ferrell, 2007; Miller and Blom, 2009; Xue et al., 2010; Trost and Kusalik, 2011; Via et al., 2011). Here we will focus on kinase-substrate interaction at the kinase domain and the substrate-peptide level, and then we will summarize the contextual information that could help to better understand the molecular determinants of kinase specificity, contributing also to boost the performances of phosphorylation site predictors.

## Inferring kinases responsible for phosphorylations *in silico*

While recent advancements in phosphoproteomics allow the identification of phosphosites from entire proteomes with ever increasing reliability and higher coverage, no high-throughput method is able to pinpoint which kinases are responsible for phosphorylating which protein substrates. Therefore, in a high-throughput context, only *in silico* approaches can effectively help in reconstructing molecular signaling circuits.

All the methods can be grouped according to different criteria, but arguably the main differences are between motif- or PSSM-based and machine learning-based methods and in the use of evolutionary information. We select seven major aspects, as exemplars of different methodologies that have been developed, namely: motif-based identification of phosphorylation sites, structural information integration, integration of phosphorylation site structural context, phospho-clusters modeling, integration of Protein-Protein Interaction Network (PPIN) information and multi-organisms prediction. For a complete list of currently available methods, see Table 1.

**Table 1:** **Computational methods for kinase-specific phosphorylation site prediction**.

**Method name**	**Approach**	**Training data**	**References**	**Website**	**Benchmark**	**Number of kinase families**	**Homology reduction**	**Accuracy**	**Cross validation**
Scansite	PSSM	OPL experimental data	Yaffe et al., 2001	scansite.mit.edu	nr^*^	62	nr	nr	nr
Scansite 2.0	PSSM	OPL experimental data	Obenauer et al., 2003	scansite3.mit.edu	nr	62	nr	nr	nr
Predikin 1.0	Structural	PhosphoBase	Brinkworth et al., 2003		nr	All kinases with structural homologs	nr	Up to 70% of known	nr
								PhosphoBase sites in top 5%	
NetphosK	Artificial Neural Network	PhosphoBase	Blom et al., 2004	cbs.dtu.dk/services/NetPhosK/	YES	6	nr	0.22–0.61 MCC depending on the kinase	3-fold
GPS	BLOSUM62 similarity + Markov Clustering	phosphoELM	Zhou et al., 2004	http://gps.biocuckoo.org/	YES	52	nr	91.8 Sn, 85.0 Sp (PKA)	nr
								94,4 Sn, 97,1 Sp (Aurora-B)	
KinasePhos 1.0	HMM	PhosphoBase + SwissProt	Huang et al., 2005	kinasephos.mbc.nctu.edu.tw/	Partial	18	nr	0.87	k-fold
PPSP	Bayesian	phosphoELM	Xue et al., 2006	bioinformatics.lcd-ustc.org/PPSP	YES	68	40–90%	90.1Sn 91.7Sp (PKA) 83.2 Sn 90.0Sp (CK2) 93.0Sn 94.1 Sp (ATM) 92.8 Sn 98.0 Sp (S6K)	Leave-one-out
pkaPS	Structural	phosphoELM	Neuberger et al., 2007	mendel.imp.univie.ac.at/sat/pkaPS	YES	1	30%	95.8 Sn 93.5 Sp (PKA)	Neighbor-jacknife
	Cluster-based	phosphoELM + MitoCheck	Moses et al., 2007		YES	1		nr	nr
KinasePhos 2.0	SVM	phosphoELM + SwissProt	Wong et al., 2007	http://KinasePhos2.mbc.nctu.edu.tw/		4	YES	91	Leave-one-out
NetworKIN	Artificial Neural Network/PSSM + PPIN context	phosphoELM	Linding et al., 2007	networkin.info	YES	222 kinases	YES	nr	YES
NetPhorest	Artificial Neural Network/PSSM	phosphoELM	Miller et al., 2008	netphorest.info	YES	222 kinases	YES	0.58–1 AUC	YES
PhoScan	PSSM	phosphoELM	Li et al., 2008	http://bioinfo.au.tsinghua.edu.cn/phoscan/		3		At high-stringency cutoffs, ~50% Sn up to 99%. Sp At low-stringency cutoffs, ~90% Sn and Sp for CDK, PKA, CK2	nr
MetaPredPS	MetaPredictor	phosphoELM + PhosphoSite + SwissProt	Wan et al., 2008	http://MetaPred.umn.edu/MetaPredPS/	YES	15	YES	0.86 (CDK)	YES
								0.89 (CK2)	
								0.85 (PKA)	
								0.78 (PKC)	
Predikin 2.0	Structural with HMM-based kinase identification	phosphoELM + SwissProt	Saunders et al., 2008	http://predikin.biosci.uq.edu.au/	YES	All kinases with structural homologs	nr	0.86–0.88 AUC (S/T)	10-fold
								0.66–0.76 AUC (Y)	
GPS 2.0	Optimized BLOSUM62 similarity	phosphoELM	Xue et al., 2008	http://gps.biocuckoo.org/	YES	70	YES	83.1 Sn 95.0 Sp (PKA)	Leave-one-out
								100.0 Sn 94.0 Sp (ATM) 78.0 Sn 95.2 Sp (CDC2) 54.0 Sn 95.3 Sp (Src)	
CRPhos	Conditional Random Fields	phosphoELM	Dang et al., 2008	http://www.ptools.ua.ac.be/CRPhos	YES	4	YES	0.93 AUC (CK2)	k-fold
								0.96 AUC (PKA)	
								0.97 AUC (CDK)	
								0.91 AUC (PKC)	
Phos3D	SVM using structural information	phosphoELM	Durek et al., 2009	http://phos3d.mpimp-golm.mpg.de/	YES	6	YES	0.73 (Ser kinases)	YES
								0.69 (Thr kinases)	
								0.67 (Tyr kinases)	
Musite	SVM	phosphoELM + SwissProt + PhosphoPep + PhosphAt	Gao et al., 2010	http://musite.sourceforge.net/	YES	13 in 6 organisms	50%	1.7–85.5 Sn, 99.9–90.0 Sp (PKA)	k-fold
								6.1–83.6 Sn, 99.9–90.0 Sp (CK2)	
								0.9–81.9 Sn, 99.9-90.0 Sp (MAPK)	
GPS 2.1	Optimized BLOSUM62 similarity	phodphoELM	Xue et al., 2011	http://gps.biocuckoo.org/	Partial	70	nr	70.73 as average on a set of kinase families	nr
PrediKin	Structural with HMM-based kinase identification	phosphoELM + SwissProt	Ellis and Kobe, 2011	http://predikin.biosci.uq.edu.au/	YES	All kinases with structural homologs	nr	0.86–0.88 AUC (S/T)	nr
								0.66–0.76 AUC (Y)	
								but lower Frobenius distance for PWMs	
RegPhos	SVM + PPI + subcellular localization	dbPTM	Lee et al., 2011	http://regphos.mbc.nctu.edu.tw/	Partial	ca. 100	YES	87.7 (PKC)	5-fold
								92.1 (PIKK)	
								92.8 (CDK)	
								91.9 (INSR)	
ConDens	Conservation of Local Motif Density	No need for training dataset	Lai et al., 2012	http://www.moseslab.csb.utoronto.ca/andyl/	YES	All kinases with known motifs	Does not apply	0.79 AUC	Does not apply
PKIS	SVM	phosphoELM	Zou et al., 2013	http://bioinformatics.ustc.edu.cn/pkis/	YES	56	YES	13.9 Sn 97.6 Sp (Erk2)	Leave-one-out
								13.5 Sn 97.3 Sp (p38a)	
								60.7 Sn (0.1 Sp (CK2 α)	
								37.5 Sn 93.3 Sp (CDC2)	
								37.3 Sn 99.8 Sp (PKCα)	
								45.0 Sn 93.0 Sp (SYK)	
								40.0 Sn 97.4 Sp (LCK)	
								23.5 Sn 94.6 Sp (FYN)	
RegPhos 2.0	SVM + PPI + subcellular localization	dbPTM	Huang et al., 2014	http://csb.cse.yzu.edu.tw/RegPhos2/		122	nr	nr	nr
	SVM	phosphoELM	Xu et al., 2014		YES	7	70%	nr	10-fold
phos_pred	Random Forest	phosphoELM	Fan et al., 2014	http://bioinformatics.ustc.edu.cn/phos_pred/	YES	54	YES	68.1–83.4 Sn, 99.1–97.2 Sp (CK2α)	nr
								68.9–82.2 Sn, 100–95.6 Sp (GSK3B)	
								71.5–83.5 Sn, 99.1–98.3 Sp (MAPK1)	
								83.8–88.0 Sn, 99.1–98.3 Sp (MAPK3) etc.	

The methods that are currently available for predicting kinase-specific phosphorylation. nr, not reported.

* *Impossible to benchmark, because no other methods were available at that time*.

The first method to predict the specific kinases that are responsible for the phosphorylations is Scansite (Yaffe et al., 2001), developed by Yaffe and colleagues, using Position Specific Scoring Matrices (PSSMs) for 62 different kinase phosphorylation motifs. Following an extensive analysis of the PKA motifs, PkaPS (Neuberger et al., 2007) was developed, exclusively suited for the prediction of protein kinase A-specific phosphorylation sites. Taking advantage of the structural information, Kobe and his collaborators developed PrediKin (Brinkworth et al., 2003; Ellis and Kobe, 2011), which is based on the analysis of the contact positions between kinases and substrates in proteins of known structure. The authors were able to associate the identification of specific kinase residues with a corresponding preference in the sequence of the substrate. PrediKin outperformed other three predictors in the DREAM4 challenge, whose goal was to predict peptide recognition domain specificity in protein kinases. In another work the information about the 3d-context of phosphorylation sites has been directly integrated in kinase-specific predictions, defining 3d-signatures motifs, even if the improvement with respect to sequence information is small (Durek et al., 2009). Conservation-based methods for predicting kinase-substrates usually assume that phosphorylation sites should be positionally conserved in Multiple Sequence Alignments (MSA) of orthologs (Budovskaya et al., 2005; Gnad et al., 2011). However, it was observed that phospho-motifs may also be found in different positions of the same local regions of orthologous proteins (Moses et al., 2007). In these cases only the local density of phosphorylation sites, but not their exact position, is conserved across orthologs. Lai et al. designed a method, ConDens, which computes the probability of observing a number of matches to a kinase motif in a MSA, under a null evolutionary model (Lai et al., 2012).

The most complete and updated collection of kinase classifiers is NetPhorest (Miller et al., 2008), currently covering 222 kinases and other fundamental signaling domains(Horn et al., 2014). Another milestone in the classification of the kinases responsible for the phosphorylations is NetworKIN (Linding et al., 2007, 2008; Horn et al., 2014), which combines the NetPhorest score with a score that considers the network context of kinases and phosphoproteins, derived from STRING (Franceschini et al., 2013) and based on genomic context, primary experimental evidence, manually curated pathway databases, and automatic literature mining.

Thanks to recent genome sequencing initiatives and phosphoproteomic efforts in several eukaryotes, organism-specific predictors have been developed (Ingrell et al., 2007; Miller et al., 2009; Gao et al., 2010). These methods aim at increasing the prediction accuracy by training on phosphopeptides derived from single organisms. The rationale for these organism-based approaches is that phosphopeptides observed in mass spectrometry experiments performed in these organisms should better represent kinome-specific phosphorylation motifs preferences (Palmeri et al., 2011).

The choice of the predictor is dramatically dependent on user needs, in terms of sensitivity and tolerance to false positives. Some predictors offer to set specific thresholds for specificity and sensitivity (Gao et al., 2010; Xue et al., 2010). Motif-based methods, depending on the motif length and distribution in the proteome, are likely to produce false positives, which can be pruned out by adding more contextual or evolutionary information. Currently, to the best of our knowledge, there is no method that takes advantage of all the aspects here reviewed. Performances will greatly vary from kinase family to family. From Src family to CDK, there are several families whose members share the same motif, and only by deploying contextual information it is possible to distinguish between those members.

Different predictors are often benchmarked using different datasets, at different redundancy levels, with different criteria, and reporting different performance measures (Table 1). Therefore, it is quite impractical to rank all the available predictors precisely, only considering the reported accuracies, and establishing the state-of-the-art is unfeasible. Initiatives, like predictors competitions such as DREAM, could be valuable opportunities to set the standards and offer more reliable evaluations.

## Kinase-peptide specificity: the kinase side

Given the relatively high frequency of Ser, Thr and Tyr residues in proteomes (in human 8.5, 5.1, and 2.5% respectively), biological systems have evolved efficient strategies to increase the signal to noise ratio and more importantly to minimize those off target phosphorylations leading to detrimental consequences.

**Figure 1:**
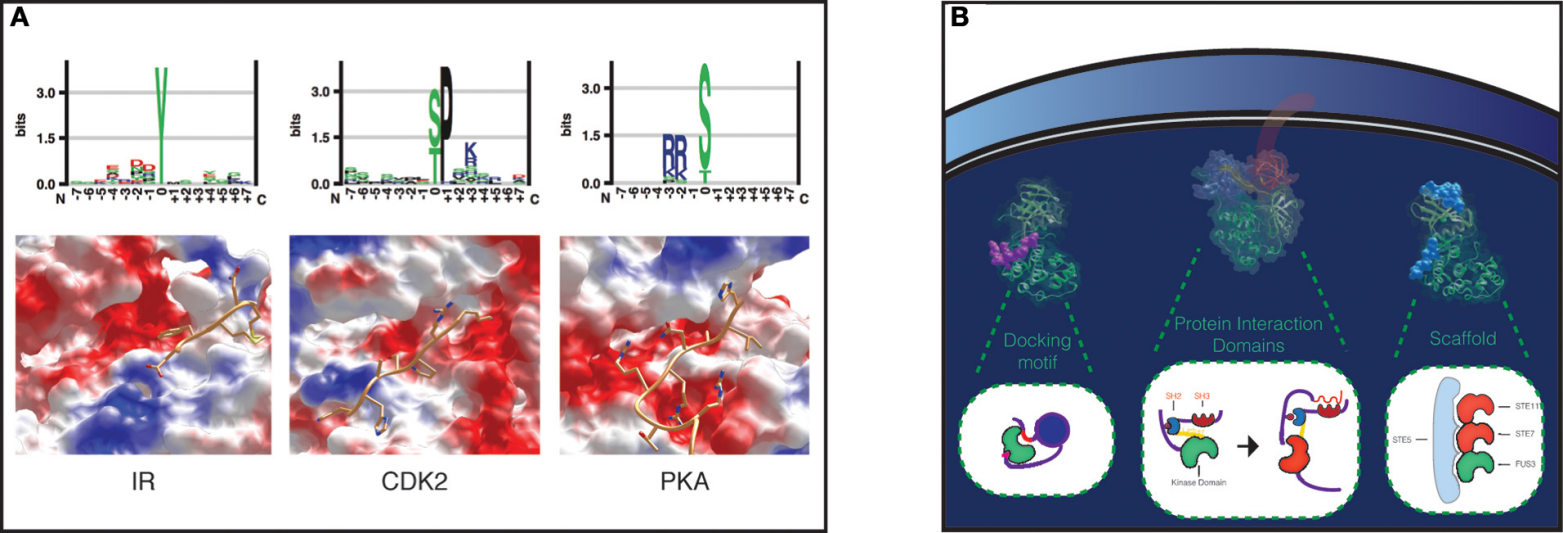
**Specificity levels in Protein Phosphorylation. (A)** Peptide specificity in a tyrosine kinase, Insulin Receptor (IR), a proline-directed kinase, Cyclin-dependent kinase 2 (CDK2), and a serine threonine kinase, cAMP-dependent protein kinase catalytic subunit alpha (PKA). Peptide preferences for each kinase are represented as sequence logos (top). The binding pockets of the three kinases have been visualized with UCSF Chimera, and the surfaces colored according to their electrostatic potential: red, positive; blue, negative; white, neutral (bottom). The structures from left to right show IR in complex with a peptide (pdb 1IR3), CDK2 in complex with a substrate peptide and cyclin A (pdb 1QMZ), which contributes to peptide specificity with a negative charged surface shown in the upper right of the figure, and PKA in complex with a peptide inhibitor (pdb 3FJQ). **(B)** Substrate recruitment. The kinase-substrate complexes concentration can be locally increased with docking motifs, protein interaction domains, and scaffold proteins. As an example of a docking motif, MAPK p38 bound to the docking site on its nuclear substrate MEF2A is shown on the left, colored in purple (pdb 1LEW). The protein interaction domains SH3 and SH2 domains in Src are fundamental for Src activation (inactive Src: pdb 2SRC), as shown in the cartoon in the middle (Xu et al., 1999). MAPK Fus3 in complex with a Ste5 peptide (pdb 2F49) is shown on the right.

The mechanisms of kinase-substrate specificity can be explored at several levels. A major separation is usually operated between peptide specificity and recruitment. Peptide specificity arises from the interactions between the catalytic kinase domain and the substrate peptide, while recruitment is based on interactions between kinase and substrate that do not involve surfaces localized at the catalytic center. During the phosphorylation reaction, the substrate is located together with the ATP in the structural region between the two kinase domain lobes, so that the gamma phosphate of the ATP can be transferred to the substrate site. The binding site differs between Ser/Thr and Tyr kinases, allowing the enzymes to discriminate between the three residues. In general each kinase shows a preference for one of these residues. Not only the site, but also its surrounding sequence provides information that is used by kinases to recognize their target sites (Figure 1A). The geometrical and electrostatic properties of the substrate binding sites across the kinases have a substantial impact on substrate specificity. Also different kinases show different electrostatic distributions over their entire surfaces that can influence substrate binding.

Usually, screenings for kinase peptide specificity are performed with Oriented Peptide Libraries (OPL) (Hutti et al., 2004). This approach revolutionized the determination of kinase specificity, using a mix of solution-phase and solid-support strategies, making kinase specificity screenings both scalable and accurate (Yaffe, 2004). It consists in the quantification of the phosphorylation frequency in degenerate peptide libraries, composed of peptides with a fixed central phosphoacceptor residue and a fixed amino acid in any one of the positions flanking the phosphoacceptor, while the remaining positions are usually drawn from a uniform amino acids distribution. The phosphorylation reaction is performed incubating the kinase with radio-labeled ATP in solution-phase, and after, thanks to a C-terminal biotin tag that is present in all libraries, the peptides are fixed to avidin-coated membranes (Songyang et al., 1994; Hutti et al., 2004; Turk, 2008). The kinase preferences for certain amino acids in fixed peptide positions can then be encoded in *consensus* sequences, in Position Specific Scoring Matrices or more complex classifiers (Miller et al., 2008). From these data, it emerges that peptide specificities of distinct protein kinases are highly variable (Ubersax and Ferrell, 2007; Turk, 2008).

It is generally assumed that the specificity between kinases and substrates is mostly driven by the substrate-binding pocket residues (Ellis and Kobe, 2011), even if also residues localized far from the kinase binding cleft may contribute to shape the peptide specificity.

## The substrate side: peptide sequence vs. 3d motifs

Durek and collegues attempted to characterize 3d-signature phosphorylation site motifs and evaluated their contribution to phosphorylation site prediction performance (Durek et al., 2009). They studied the spatial distribution of amino acids from 2 to 10 Angstrom around each phosphosite, and defined family-specific 3d-profiles. They reported a modest improvement in predicting the kinase families that phosphorylate serine phosphosites, due to the inclusion of structural information. Despite the small discriminatory power of 3d motifs, structural information, like disorder and secondary structure predictions can more efficiently be deployed to improve phosphorylation site predictors performances (Iakoucheva et al., 2004; Durek et al., 2009).

## The substrate side: peptide interpositional dependence

In 2012 Joughin et al. explored the inter-positional dependence on substrates of ATM/ATR, Cdk1/Cyclin B and CK2 kinases (Joughin et al., 2012). They found only a few significant substrate sequence position pairs that show deviations from position-wise independence. They also tested the ability of first and second order models to correctly separate between the true kinase substrates and mock substrates. Firstly they just used shuffled negative controls (i.e., they shuffled the substrate peptide positions, drawing from the distribution of the true substrates in each position), and they uncovered that mock substrates were similar in quality to the true ones. Then, by using proteomically derived mock substrates, they uncovered that second order were either equal to first order models, or due to over-fitting in training, even worse. Therefore, they concluded that higher-order interdependences in peptides do not seem to give a significant contribution to predictive performances.

This work has interesting implications for the evolution of signaling networks. There are several examples in the literature showing that the molecular recognition of substrates and phosphopeptide-binding domains is subjected to inter-positional dependences. If also other kinases not included in this study turn out not to show marked second or higher-order preferences on substrate sequences, this would mean that there is a fundamental difference in the way these two components behave in the evolution of signaling networks. As the authors point out, the fitness landscape might look smooth for kinase peptide specificity, and the fitness of the kinase substrate could be boosted after sequential mutations on the peptide, while the fitness landscape for phosphopeptide-binding domain substrate may contain energetic barriers. From a phosphosite predictor perspective, this means that greater efforts should be centered around the development of context-dependent methodologies.

## Placing kinase-peptide specificity in context

Although modeling kinase-peptide specificity is fundamental for understanding kinases preferences for their substrates, to study signal propagation in biological systems all phosphorylation events need also to be placed in time and space. As Alexander and colleagues clearly demonstrated in a paper published in 2011, the *in vivo* specificity of mitotic kinases arises from both subcellular localization and preferences for phosphorylation motifs (Alexander et al., 2011). For the first time they described an evolutionary conserved mechanism based on a combination of negative and positive phosphorylation motifs selection and spatial localization, to secure proper signal propagation during mitosis. Thus, even if two kinases can share a phosphorylation motif or can localize in the same place, none of the mitotic kinases shares similar preferences in phosphorylation motifs and is also co-localized with any other mitotic kinase.

## Substrate recruitment

Protein kinases are highly flexible molecules, and this intrinsic flexibility has likely favored the engineering of complex regulatory and specificity mechanisms throughout eukaryotic evolution. Several mechanisms of recruitment are peculiar to some kinase families, and they can generally be grouped into: scaffold interactions, docking sites, and domain-domain interactions (Reményi et al., 2006; Miller et al., 2008) (Figure 1B).

Scaffold proteins can contribute to specificity increasing the local concentration of the kinase and the substrate, thus enhancing phosphorylation. Probably the best known are MAPK and PKA scaffolds (Wong and Scott, 2004; Strickfaden et al., 2007).

**Figure 2:**
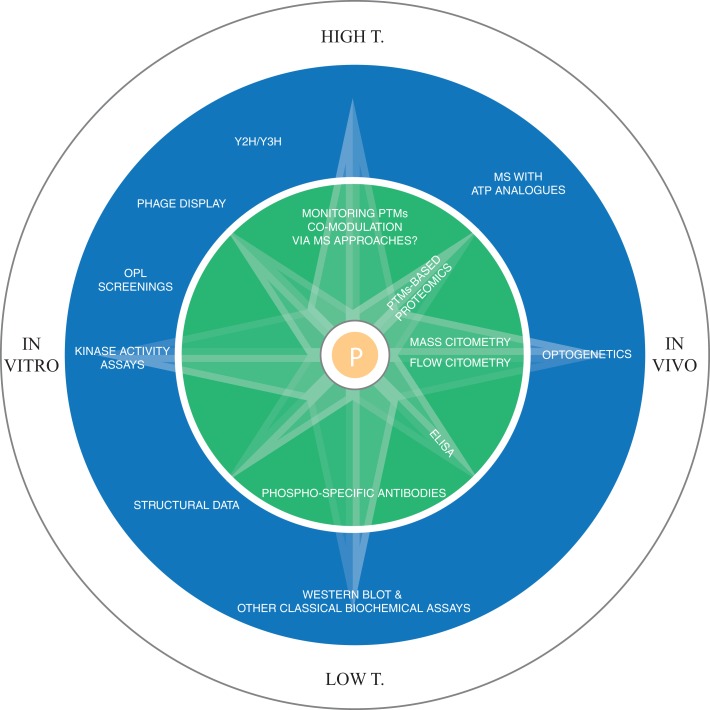
**Methodologies for the experimental identification of phosphorylation sites**. In the inner circle the techniques for the detection of phosphorylation sites are reported, while the outer circle displays the major techniques for dissecting kinase-substrates interactions, both at the level of direct determination of kinase-substrate interaction (kinase activity assays, OPL, MS with ATP analogs, structural data, western blot, optogenetics) and at the contextual information generation level, i.e., the methodologies that allow the identification of interacting domain preferences, domain-peptide interactions, etc. (Y2H, Y3H, phage display, structural data, western blot, optogenetics). In the field of PTM identification, future advancements in MS will allow the monitoring of multiple PTMs co-modulation, while for kinase-substrate interactions, the use of ATP analogs coupled with MS/MS is currently the most promising high-throughput technique to link kinases to their substrates *in vivo*. Y2H/Y3H: yeast 2/3 hybrid system (Y3H could be deployed for the study of scaffold proteins-mediated interactions).

Docking motifs are distant from the phosphosite and facilitate the kinase-substrate recognition (Biondi and Nebreda, 2003). They can be discovered using experimental screening of focused or randomized peptide libraries (Reményi et al., 2006). In the case of Tyr kinases, the motif is usually found in domains that are different from the ones that catalyze the phosphorylation reaction. The motif can also be induced, as in the case of conditional docking sites, where the kinase is recruited only after a phosphorylation event takes place in the motif (Elia et al., 2003). This could also be a way used by the cell to implement logic gates and keep the timing of phosphorylation. Protein interaction domains, like SH2, SH3, PTB, 14-3-3 can also promote the association between kinases and substrates. Src activation, for instance, is mediated by its SH2 domain (Xu et al., 1999) (see Figure 1B). Domain-peptide interactions can be studied experimentally with peptide binding assays, while domain-domain interactions can be modeled using data collected in several databases (Luo et al., 2011; Yellaboina et al., 2011; Kim et al., 2012; Mosca et al., 2014), from high-throughput experiments, like yeast two hybrid, or extracted from the literature (see Figure 2 for an overview of the major experimental techniques used to identify phosphorylation sites and kinase-substrates interactions).

In 2011 Won and colleagues described the contribution of recruitment interactions to the kinase specificity of Ste7, a MAPKK involved in mating signal flow in *S. cerevisiae* (Won et al., 2011). Ste7 has an interaction with the scaffold protein Ste5 and two docking interactions that allow it to bind to the MAPK Fus3. They uncovered that two out of the three other MAPKK encoded in *S. cerevisiae* genome can functionally replace the MAPKK Ste7, grafting recruitment interactions in their kinase domain. Notably, grafting only the scaffold, or only the docking interactions is not enough to restore the mating signal. This underlines the critical importance of recruitment mechanisms acting concertedly. Scaffold proteins mediating interactions may in theory be discovered using a yeast three hybrid approach, where the kinase and the substrate are fused as bait and pray, and their indirect interaction could be tested expressing the scaffold.

The cellular context has a fundamental role in the determination of the substrate specificity. For instance, kinase localization is important for proper CDKs function. A number of cyclins activate and localize CDKs to different compartments. Overexpression of cyclin B1 causes chromosome condensation, reorganization of the microtubules, and disassembly of the nuclear *lamina* and of the Golgi *apparatus*, while overexpression of cyclin B2 only causes the disassembly of Golgi *apparatus*. Changing the localization motifs, and swapping the two cyclins localizations reverses their phenotypes (Draviam et al., 2001).

Several mechanisms of specificity have recently been explored also for protein phosphatases (Tiganis and Bennett, 2007; Roy and Cyert, 2009). Despite these enzymes lack strong preferences for substrate sequences, higher specificity is obtained with recruitment *via* domains and short linear motifs-mediated interactions and subcellular localization (Sacco et al., 2012).

## Properties and evolution of post-translational regulatory networks

Currently tens of thousands of phosphorylation sites can be identified by MS-based proteomics in a single experiment (Olsen and Mann, 2013). These large-scale datasets challenged the view of PTMs gained from low-throughput experiments, where a few highly important sites are studied, questioning the functionality of all these PTMs. Several evolutionary studies on phosphorylation sites have confirmed that sites known to be associated to a function are significantly more conserved than non-phosphorylated residues (Gnad et al., 2007; Malik et al., 2008; Landry et al., 2009; Tan et al., 2009a; Moses and Landry, 2010). However, a large fraction of phosphorylation sites identified in high-throughput experiments does not show strong evolutionary conservation.

The high evolutionary turnover of phosphoproteomes may be due either to non-functional phosphorylation sites or to species-specific regulation. Recently, a model has been proposed that could explain observations about phosphorylation enrichment in abundant proteins combined with the low stoichiometry of these phosphorylation sites (Levy et al., 2012; Tan and Bader, 2012). According to this model, random encounters between a kinase and proteins in the same subcellular location could end up in a-specific phosphorylations, that will more likely affect highly abundant proteins. This model implies also that only a minimal fraction of an abundant protein population should host these off-target sites. Not all *unintended* phosphorylations are necessarily damaging the cell, otherwise they would have been removed during evolution. Therefore, a fraction of all phosphorylation events could be neutral from an evolutionary perspective. In this *scenario*, the upper bound to the accumulation in the proteome of such sites during evolution is the signaling networks tolerance to noise levels. A nice example of noise minimization has been observed in metazoan lineage evolution, where it has been hypothesized that the signaling networks may have eliminated detrimental phosphorylation sites and limited the noise in the system as tyrosine kinases expanded, by tyrosine-removing mutations (Tan et al., 2009b). In shorter evolutionary distances, the signaling networks properties will more tightly be coupled with the mutational properties of the codons encoding the different phosphorylatable residues. Amongst all residues, serine is considered a mutational hub, as it is very close in mutational space to most residues (Creixell et al., 2012). Indeed it is the only amino acid whose codons are distributed in two groups that are at least two mutations away from each other.

Another explanation for the low conservation of some phosphorylation sites could reside in compensatory mechanisms. In a pioneering work Bodenmiller and colleagues performed single deletions of all yeast kinases and phosphatases, surprisingly observing only a small amount of regulation in the phosphoproteome (Bodenmiller et al., 2010). Even more strikingly, the indirect effects are predominant on effects on the direct targets of the deleted kinases, without strong phenotype alterations, in agreement with the view of signaling networks as systems that are robust to perturbations. Crucially this highlights that a similar cellular state can be the result of different systems regulations. From an evolutionary perspective, different signaling solutions, independently evolved, could be analogous implementations of the same function.

A consistent fraction of eukaryotic phosphoproteomes may represent an evolutionary reservoir that the different organisms could exploit to evolve specific regulation. Estimating more precisely the magnitude of these *non-functional* phosphorylations will contribute in the near future to improve our understanding of post-translational regulatory networks and their properties.

In the case of phosphosites involved in modulating protein-protein interactions, the site may not necessarily be positionally conserved (Tan et al., 2010). Phosphosites in different organisms at the same interface tend also to be phosphorylated by kinases of similar specificity. Therefore, the same protein interface may be modulated by functionally redundant sites that are weakly conserved in sequence (Tan et al., 2010; Palmeri et al., 2014).

Many domains in the human proteome seem to have peculiar preferences for being targets of phosphorylation. Some domains, like the kinase domain, tend to be significantly enriched, while other ones tend to be depleted in phosphorylation. Within the same domain, phospho-hot spots can also be identified, i.e., regions that are highly enriched in phosphorylation, suggesting modulation of the domain function via these segments, as in the kinase activation loop, or the C-terminus of HSP90 domain (Beltrao et al., 2012). The domain context of a phosphosite can then be used to functionally characterize the site, and also to improve phosphosite predictor performances (Palmeri et al., 2014).

## PTMs cross-talk

Several low-throughput experiments offer nice examples of how the cell uses PTMs combinations to reach highly sophisticated levels of control (Lo et al., 2001; Choudhary et al., 2009; Wang et al., 2011; Zheng et al., 2011). Difficulties in high-throughput determination of co-modulation between different PTMs currently limit the scale at which analysis of cross-regulation can be conducted. However, in a recent work, Swaney and colleagues studied the cross-talk between phosphorylation and ubiquitylation after proteasome inhibition, thus identifying potential phosphodegrons, analyzing the pairs of phosphorylation sites and ubiquitylation sites that increased in abundance after proteasome inhibition (Swaney et al., 2013). Computational works have already started exploring this relatively novel territory. For instance, Woodsmith and collaborators suggested that PTMs clusters may represent signal integration platforms (Woodsmith et al., 2013). In another work, Minguez and colleagues from Bork's group, used the concept of correlated evolution to discover new types of co-regulation within different PTMs (Minguez et al., 2012). Greater efforts in the developments of experimental methods for large scale monitoring of co-modulated PTMs will enormously help in understanding how the signaling networks respond to and integrate different inputs from a systems level perspective. But also new computational models will have to be developed and may benefit from coordinated modeling of the different PTMs.

## Conclusion: kinase-substrate specificity modeling

Modeling kinase specificity for substrates is one of the most challenging bioinformatics contributions to cellular signaling. Mass Spectrometry is able to generate large amount of data, but there is currently no high-throughput experimental way to identify the candidate responsible for the phosphorylation.

The two main challenges in developing computational approaches to kinase-substrate specificity are: modeling kinase-peptide specificity and substrate recruitment preferences. Bioinformatics solutions have extensively explored the motif-context, and as pointed out by Joughin et al. the construction of higher order mathematical models might have limited, if any, advantage, at the high cost of overfitted models (Joughin et al., 2012). *In silico* modeling efforts should be centered around a more effective integration of different levels of contextual information, placing the kinases and the substrates in correct space and time, but also considering the interactions outside the kinase domain (domain-domain, scaffolds and docking sites interactions), that can increase locally the concentration of kinases and substrates. Data is obviously critical to all this, therefore more OPL screenings on kinases with unknown specificity, combined with more comprehensive studies to explore scaffold-mediated kinase-substrate interactions and also more efforts dedicated to assess the impact of mutations in domain-domain interactions involved in signaling could contribute to the development of more refined models of kinase-substrate specificity. Probably the most difficult challenge is to convert results from *in vitro* studies to approaches that make reliable *in vivo* predictions. Different kinases vary in their dependence on contextual information. Holistic, i.e., highly integrative, approaches, that allow the modeling of many contexts at the same time, will be able in the future to dissect for each kinase (or kinase group) the different contributions that shape the logic of the signaling system, like for instance in the remarkable case of mitotic kinases, studied by Yaffe's group (Alexander et al., 2011). High-throughput identification of the kinases responsible for the phosphorylation events is critical to achieve this. MS coupled with ATP analogs is currently the most promising approach in this field (Lopez et al., 2013).

Function-dependent classifiers, like those considering the identity of the domain where the phosphorylation is located, can be an alternative way to boost performances in phosphorylation prediction and they might be considered also for biomedical applications. Lastly, from the computational integration of different PTMs models (Creixell and Linding, 2012; Minguez et al., 2012), it may be possible, in the future, to infer and monitor system-level regulatory centers, whose function might be impaired in complex diseases.

### Conflict of interest statement

The authors declare that the research was conducted in the absence of any commercial or financial relationships that could be construed as a potential conflict of interest.
